# The syntactic organization of pasta-eating and the structure of reach movements in the head-fixed mouse

**DOI:** 10.1038/s41598-017-10796-y

**Published:** 2017-09-08

**Authors:** Ian Q. Whishaw, Jamshid Faraji, Jessica R. Kuntz, Behroo Mirza Agha, Gerlinde A. S. Metz, Majid H. Mohajerani

**Affiliations:** 1 Department of Neuroscience, Canadian Centre for Behavioural Neuroscience, University of Lethbridge, Lethbridge, AB T1K 3M4 Canada; 2 Golestan University of Medical Sciences, Faculty of Nursing & Midwifery, Gorgan, I.R. of Iran

## Abstract

Mice are adept in the use of their hands for activities such as feeding, which has led to their use in investigations of the neural basis of skilled-movements. We describe the syntactic organization of pasta-eating and the structure of hand movements used for pasta manipulation by the head-fixed mouse. An ethogram of mice consuming pieces of spaghetti reveals that they eat in bite/chew bouts. A bout begins with pasta lifted to the mouth and then manipulated with hand movements into a preferred orientation for biting. Manipulation involves many hand release-reach movements, each with a similar structure. A hand is advanced from a digit closed and flexed (collect) position to a digit extended and open position (overgrasp) and then to a digit closed and flexed (grasp) position. Reach distance, hand shaping, and grasp patterns featuring precision grasps or whole hand grasps are related. To bite, mice display hand preference and asymmetric grasps; one hand (guide grasp) directs food into the mouth and the other stabilizes the pasta for biting. When chewing after biting, the hands hold the pasta in a symmetric resting position. Pasta-eating is organized and features structured hand movements and so lends itself to the neural investigation of skilled-movements.

## Introduction

Skilled hand use by rodents, including the mouse, have been described in tasks of skilled reaching, in which an animal extends a hand to grasp a piece of food to place it in the mouth for eating^1–4^. These studies show that the behavior is organized, in that the animal orients to the target, localizes the target using olfaction, and then reaches to grasp the food to place it in its mouth for eating. Reaching movements are structured with proximal upper arm/shoulder movements that aim, extend, pronate, supinate, and withdraw the hand and distal hand movements that shape the digits, grasp, and release the food into the mouth. Beginning with Kalil and Schneider’s^5^ description of sunflower seed holding by hamsters with unilateral pyramidal tract lesions, there have been a number of descriptions in rodents of hand postures used when spontaneously handling food for eating. Food holding postures are different in different species of rodents^6^ and the complexity of holding postures varies depending upon whether an animal is handling a prey item such as a cricket^7^, nuts that require shell removal, or food items such as pasta of various types that require complex manipulation^8^. These studies find that not only do animals vary their arm postures as a function of food type, they also vary their hand preshape and grasp movements in relation to food size as food size becomes smaller as it is consumed^6, 9^. A number of rating methods for static food holding postures have been found useful for detecting hand use deficits in models of nervous system disease including stroke, Parkinson disease, Huntington disease, and spinal cord injury^10–13^.

With the development of head-fixed procedures that facilitate the concurrent monitoring of skilled movements with electrophysiology, *in vivo* imaging, and optogenetics, there is interest in investigating the motor cortex’s forelimb region’s contributions to behavior using mesoscale methods^14–18^. In a previous study we have also observed that in the course of eating pieces of pasta, their hand grasps vary depending upon where they grasp the pasta^19^. Nevertheless, most of the behavioral observations in both free-moving and head-fixed mice during pasta eating, are descriptions of the static features of pasta holding and are mainly developed for detecting abnormalities that may signify brain injury^9–13^. To date, there has been no comprehensive description of the dynamic features of pasta eating; that is, the organization of movement and the structure of individual acts, for either free-moving or head-fixed mice. There are three reasons why a description of the dynamic features of pasta eating would be useful. First, for online mesoscale monitoring of neural activity a description of the dynamic features of hand movement could be useful. Second,were mice able to display organized behavior when eating pasta, the task would be of special interest because head-fixed animals are extremely limited in their ability to display other organized behaviors that depend upon neocortical function, such as nest building, sexual, or play behavior. Third, a head-fixed procedure for examining hand movements is especially useful because the hand area of the motor cortex comprises a large area of motor cortex and is located on the dorsal frontal cortical surface where it is readily accessible for stimulation and imaging procedures during concurrent behavior^20–23^.

The purpose of the present study was twofold: to describe the overall organization of pasta eating and to describe the structure of the forelimb movements used for manipulating pasta for head-fixed mice. In the experiment, mice reached for short pieces of pasta and their hand movements as they ate the pasta were filmed using high-speed videography and analyzed by frame-by-frame video analysis. Attention was also directed to the overall pattern of hand use by using an ethogram of eating behavior with subsequent descriptions of the behaviors displayed in the ethogram, including details of reaching and grasp patterns of pasta manipulation. The analyses indicate that the behavior of pasta eating is organized and that the mice use a structured reach that is modified online as required for pasta manipulation.

## Behavioural Results

### Reach success

All 9 mice successfully reached for and ate pasta with a mean success of 67 ± 5%, of the 10 pieces of pasta for which they reached^19^. Because every mouse successfully obtained and ate 4 pieces of pasta, the present results are based on the analysis of the first four pieces of pasta consumed by each mouse.

### Patterns of pasta holding

As an overview, the mice displayed two general types of hand grasps on the pasta, as has been previously described^9–13, 19^. Support grasps involved a hand grasping the pasta by wrapping two or more digits around it, with additional support from the palm. Guide grasps involved contacting the pasta with the tips of one or more digits, mainly the tip of digit 2. Support grasps were mainly directed to the distal end of the pasta, relative to the mouth. Guide grasps were mainly directed to the proximal end of the pasta, relative to the mouth. One or both hands could make support grasps at the same time, but generally only one hand made guide grasps as the other made a support grasp. When a mouse reached for the pasta to make a support grasp they seldom missed the pasta, but when a hand was making guide contacts with the pasta, many of the reaches to the pasta ended without contact. The reason for these noncontact movements is uncertain, but they may be related to the fact that when support hand held the pasta at an angle facing away from the guide hand, the pasta protruded from the opposite side of the mouth and so was difficult to contact on the side of the mouth facing the guide hand. A count of the grasp type used was made of each time that a hand released the pasta and then renewed contact it.

**Figure 1:**
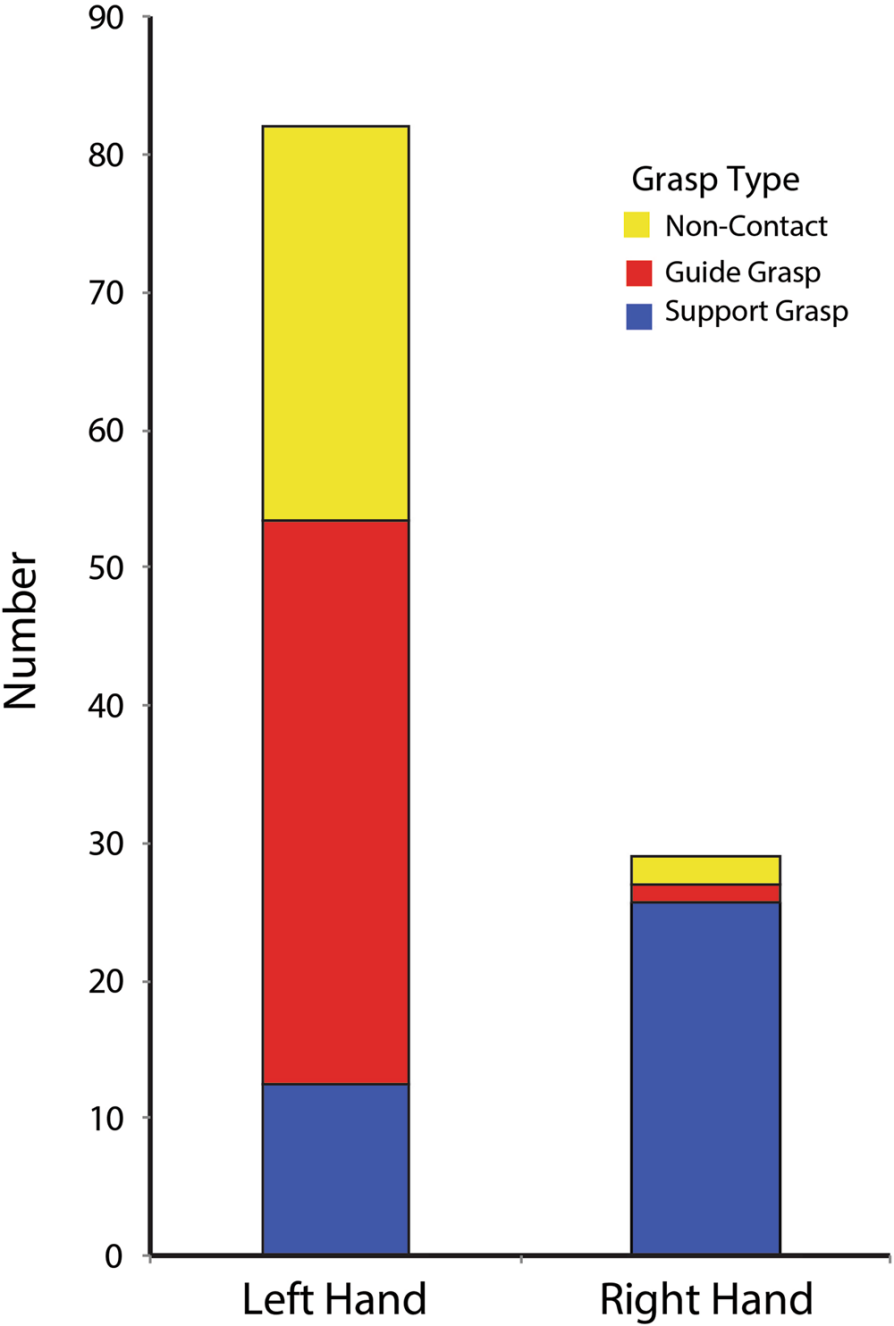
Grasp frequency and asymmetry. Average grasp type made by the right and left hand by one mouse eating four pieces of pasta. Each grasp was defined as releasing and then repurchasing the pasta. Note: more support reaches are made by the right hand and more guide reaches are made by the left hand. Note the many left hand movements in which the hand was advanced to the pasta but did not touch it.

The average counts of grasp types for one mouse consuming four pieces of pasta is shown in Fig. 1. It is noteworthy that the grasp type used by left and right hand is asymmetric, with many more support grasps made by the right hand and many more guide grasps made by the left hand. In addition, the left hand making guide grasps displayed many more reaches, both for contact and for noncontact, than are displayed by the hand making support grasps. Because the mice varied in their asymmetry (see below) a representation from one mouse is a more accurate description of its behaviour than averaged group data would be. Nevertheless, the average number of miss reaches per piece of pasta for all mice was: left hand, 8 ± 0.97, right hand 11 ± 4.5; the average number of guide reaches were: left hand 6 ± 3.07, right hand 22.55 ± 7.11; and the average number of support reaches were: left hand 20.44 ± 3.07, right hand 18.77 ± 3.12 (n = 9 mice).

### Ethogram of reaching and eating

A representative ethogram derived from a single mouse eating its first piece of pasta is shown in Table 1. Observation of the mouse pasta handling movements indicated that the mouse engaged in a complex series of actions in which it positioned the pasta in its mouth to bite off pieces of pasta, while periodically adjusting the pasta by removing its hands from the pasta and reaching for it again so that its hands were located in a new position on the pasta. Once a mouse succeeded in biting off a piece of pasta it lowered its hands and the pasta, and chewed. It resumed manipulatory and biting actions when chewing was complete. The ethogram indicates that pasta eating movements are organized and can be subdivided into bouts of biting and chewing, with biting accompanied by many right and left hand movements to orient the pasta. When the ethogram analysis was applied to the same mouse eating other pieces of pasta, and applied to other mice, (n = 9 mice) whether eating duration was long or short, it was found to provide a good representation of the mouse pasta-eating action. In the following sections, components of the behaviour derived from the ethogram are described.

**Table 1:** Ethogram of hand movements for eating one piece of pasta. Ethogram initiated when pasta enters mouth (I). All subsequent behaviors are recorded in bouts, which are recorded on a single line. Bouts begin when the pasta is removed from the mouth (0). Hand shapes made by the left and right hand are rated on a 3 point scale (R,R,r/L,L,l) in the order of occurrence.

I	L R L R		
O c I	L R L-R l r l L-R		
O c I	l L L L-R Rm r L r r R R r r r r r R R R	I	pasta into mouth
O c I	R L R R r R R R rm R R R R r r r r R R R r r r R R	O	pasta out of the mouth
O c I	R R R-L r r Rm r r R	C	chew
O c I	L-R r L L-R R L R-L R-L	R	right open hand
O c I	L r L L-R L L R L-R R R R	R	right partially closed hand
O c I	L-R L L-R L r r r R R R r R R R R r r r r r R r r R r r	r	right closed hand
O c I	L-R R R r	L	left open hand
O c I	r-Lm Rm L-R L-R Rm R	L	left partially closed hand
O c I	L Rm R r r r	I	left closed hand
O c I	R Rm Rm R	L-R	both hand
O I	R R R r r r r r	m	miss
O c I	L-R L-Rm R-L R r		
O c I	L L L R-L R-L R	Total Time	1:15 min

#### Eat bouts

Each line in the ethogram illustrates an eating bout (in this example 15 bouts in total). A bout consisted of holding a piece of pasta to the mouth and manipulating it so that a piece of pasta could be bitten off, following which the pasta was removed from the mouth while the mouse engaged in chewing. Thus, each bout begins by inserting (I) the pasta into the mouth after chewing (C) the previous piece. For this representative mouse, 75 sec was spent holding and handling the pasta for eating and 39 sec was spent with the pasta removed from the mouth while the mouse chewed. For all of the mice eating 4 pieces of pasta, the mean and standard error for the number of bouts was 6.4 ± 0.6. The mean and standard error for manipulate and bite time for a piece of pasta was 32.56 ± 4.5 sec, and for chewing time it was 13.8 ± 1.56 sec (n = 9 mice).

#### Reaching

When manipulating the pasta in the mouth to position it to bite off a piece of pasta, a mouse made many movements of releasing a hand from the pasta and then regaining its grasp. Each repositioning movement by either the right or left hand thus comprised a reach. Reaches are indicated on the ethogram by symbols for the left (L) and right (R) hand. The mouse illustrated in the representative ethogram (Table 1) made 191 reaches (all reaches by either the right or left hand). In other words, in terms of the animal’s eating duration of 75 sec, one or both hands released the pasta and reached to reposition it on the pasta 2.54 times per second. The average number of reaches made by all mice was 80.33 ± 12.70 and their eating duration was 20.77 ± 2.9. Thus, the average number of times a mouse repositioned its digits was 3.86 time per second (n = 9).

#### Hand preshaping

During a reach, and before the hand grasped the pasta, the hand was preshaped in relation to the type of grasp the hand was to make. Hand preshape could be relatively closed with the digits partially closed and flexed, with the digits partially extended and open, or with the digits fully open and extended. In the ethogram (Table 1), the relatively closed preshape for the left or right hand is designated as “l or r”, the open preshape is designated as “L or R” intermediate preshapes are designated as “L” or “R”. If an attempted reach contacted the pasta but did not grasp it, the reach is indicated by “m” for miss.

#### Support and guide grasps

An examination of the relation between hand shaping and the type of grasp made with the pasta indicated that large hand preshapes usually made whole hand grasps that supported the pasta whereas smaller hand preshapes usually formed guide grasps that guided the pasta into the mouth.

**Figure 2:**
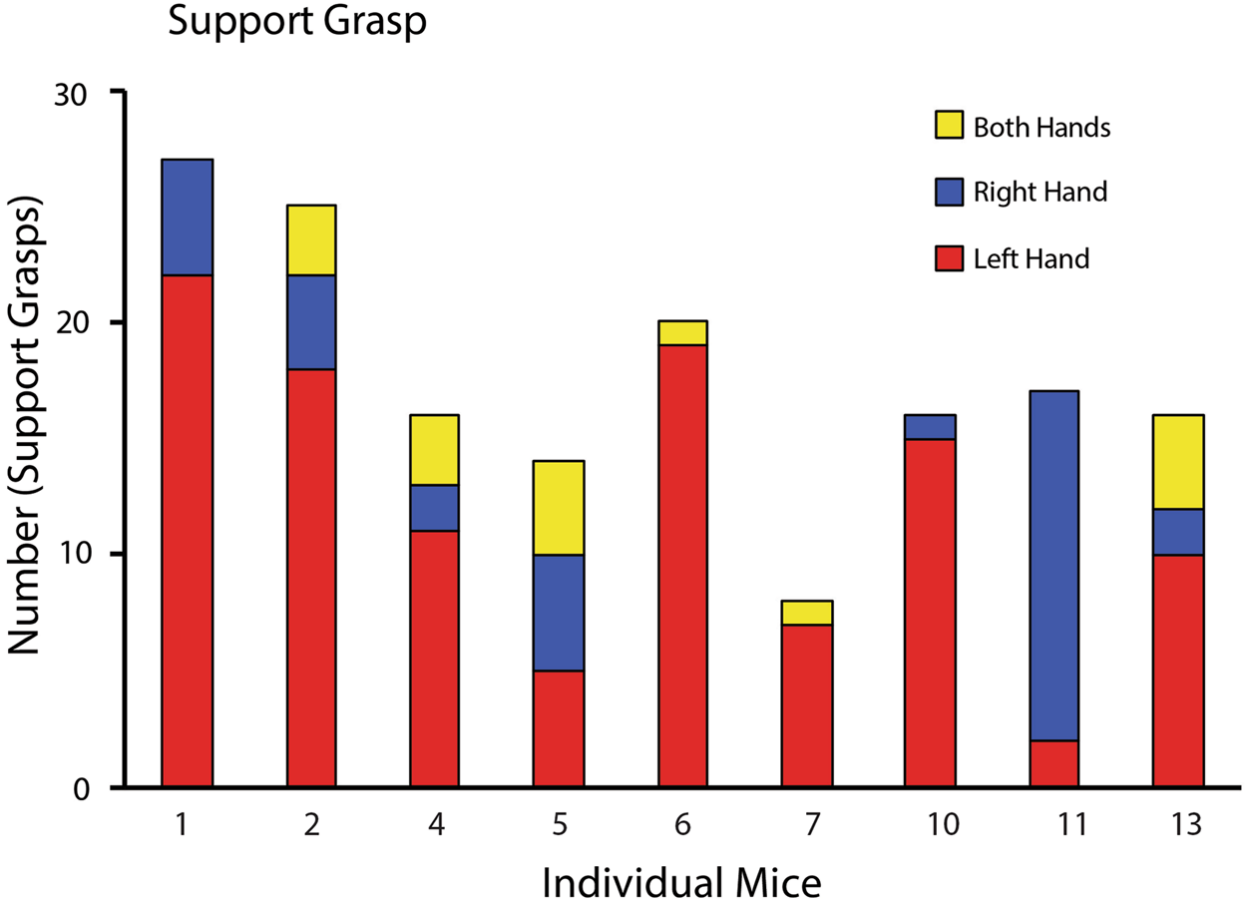
Support grasp frequency when eating. Number of separate occurrences of support hand use in each eating bout for four piece of pasta in individual mice. Seven mice displayed a left hand preference, one a right hand preference, and one no clear preference in the hand used for support.

Because support grasps were preferentially made by one hand and so were indicative of an asymmetric behaviour displayed by each mouse, counts were made of the number of times that each hand was used as the support hand for each bout of eating by each mouse. Figure 2 illustrates: (1) the average number of support grasps varied in different mice from less than 10 to more than 20, and (2) most of the mice displayed varying degrees of preference for using one hand as the support hand. Seven of the 9 mice preferred to use the left hand as the support hand, one mouse (#5) alternated support hands, one showed no preference (mouse #4), and one preferred the right hand (mouse #8) as the support hand. As the mice were trained to use the left hand to reach, it is uncertain whether this training influenced their preference of which hand they used to support the pasta. In addition, occasionally, some of the mice used both hands in a support role.

### Hand preference and pasta orientation for biting

Many of the reaching movements made by the mice were directed toward manipulating the pasta into a position from which a mouse could bite a piece from it. The pasta was repositioned by removing the support hand from the pasta, thus allowing the pasta to be moved by the mouth, or the other hand, to a new position. When the support hand was removed the guide hand might remain on the pasta or it might be removed along with the support hand or independently of the support hand. A number of hand removal, reposition, and reach movements might occur before the pasta was positioned for biting.

**Figure 3:**
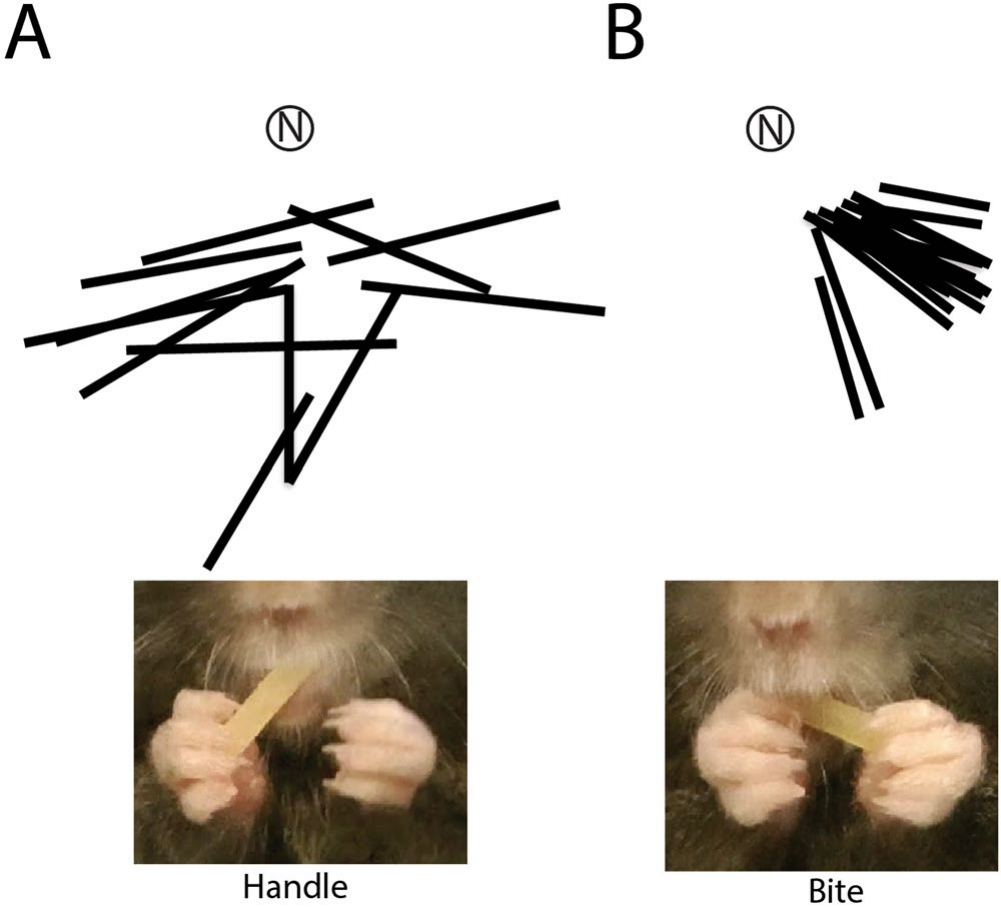
Manipulating and biting orientation. (**A**) Location of the pasta each time it was released by the support hand during one manipulatory sequence. (**B**) Pasta orientation for the execution of a bite for all bites by one mouse eating one piece of pasta. Note: manipulation brings the pasta to the preferred orientation for biting. N, nose.

An example of a manipulatory sequence from one mouse is illustrated in Fig. 3A. In this figure, the pasta is first supported in the right hand, which was not the preferred support hand of this mouse. The successive orientations of the pasta are those on which the left hand released the pasta between each of its successive reaches. Between reaches the pasta moved to a new position largely as a result of tongue and mouth movements but sometimes also in response to pressure exerted by the right hand. The reorienting sequence ends when the left hand has a supporting grasp allowing it to hold the pasta for biting.

Figure 3B shows the pasta’s orientation when it was positioned for biting for each bite for the same mouse. The preferred orientation at the time of the bite, which was on average 54° to the left of center for this mouse (relative to the mouth), i.e., all bites occurred when the pasta located in the lower left quadrant relative to the mouse’s mouth. Bites were made with the molar teeth on the opposite side the pasta’s orientation. An analysis of pasta orientation by quadrant for all bites by all of the mice gave a significant effects of quadrant, F(1,3) = 10.27, p < 0.001 (n = 9). The significant effect occurred because preferred orientations were either in the left or right lower quadrants. A correlation between preferred quadrant of pasta orientation and the hand preferred for supporting the pasta for all mice was r = 0.89, p < 0.001 (n = 9). Thus, each bite instance occurred with the pasta oriented at about the center of the left or right lower quadrant relative to the preferred support hand. This orientation presumably provided a good point for pushing down on the pasta to break off a piece for subsequent chewing. Even on the few instances when a mouse supported the pasta with both hands and bit off pieces from the middle, the pasta was oriented on a similar angle to that used when the pasta was supported by one hand.

### Grasp patterns and digit use

In order to quantify digit participation in grasping the pasta, digit contacts with the pasta were recorded on each of the first 1,000 video frames of pasta eating that were made by 4 mice. Measures began as the mice first placed the pasta in the mouth, but did not include periods when the mice were chewing. Digit contacts were recorded by shading in a set of circles that represented the digits 2–5 of the left hand and digits 2–5 of the right hand for each video frame. The results were summarized as the number of frames on which each digit combination was in contact with the pasta.

**Figure 4:**
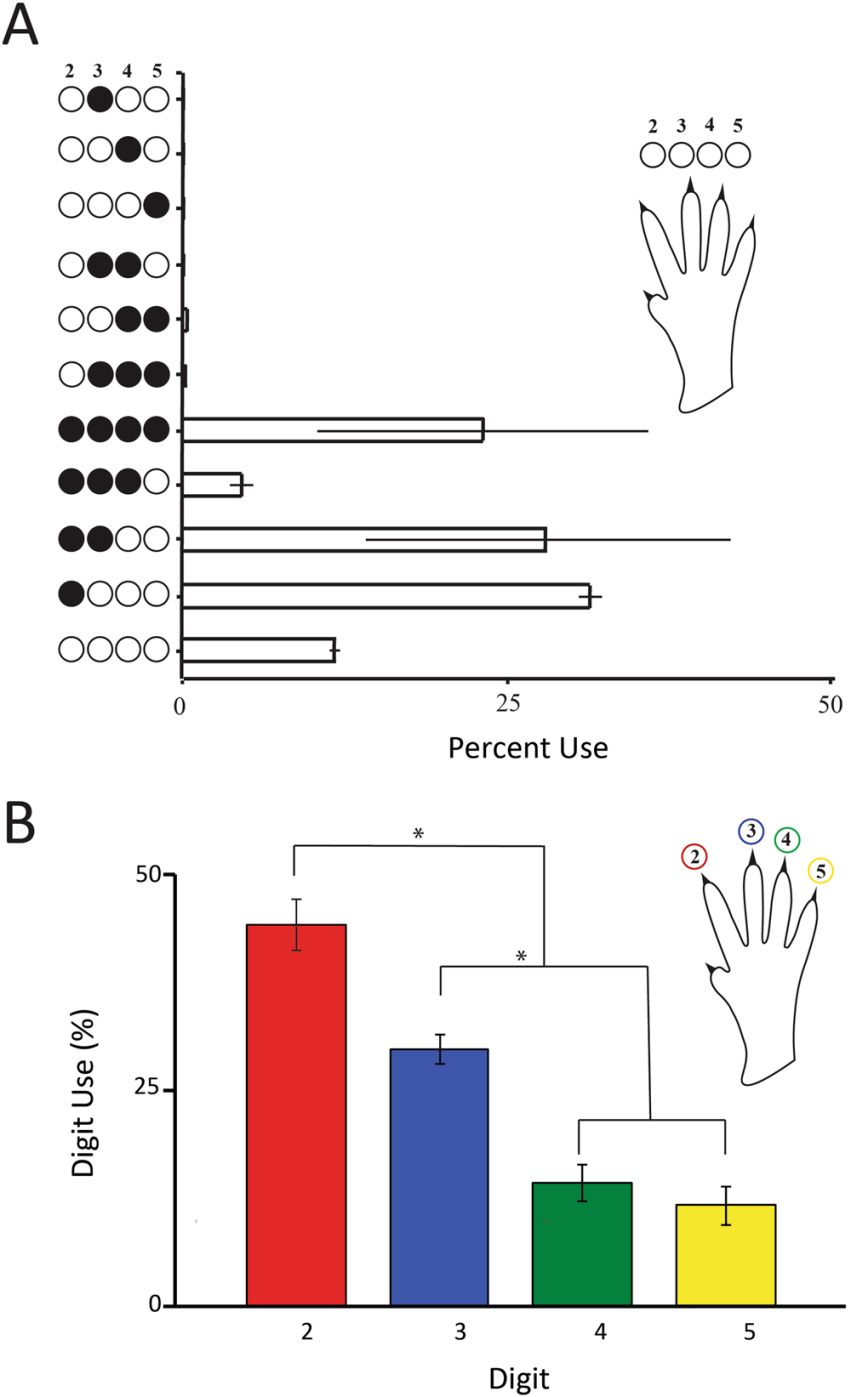
(**A**) Digit use for holding pasta (mean percent) obtained from 1,000 video frames for each of four mice. Mice used three main digit patterns for holding pasta: (1) digit 2 to guide the pasta into the mouth, (2) digit 2–3 to support the pasta, and (3) digits 2-5 to support the pasta. (**B**) Frequency of digit use for pasta holding. Digit 2 was the most frequently used digit for holding (p < 0.001, n = 9). When no digit contact is indicated, the pasta if being held by the mouth.

A summary of the results of digit contact patterns in terms of percent of frames on which contact combinations were made is shown in Fig. 4A. The results indicate that a relatively small number of digit patterns accounted for most of the contacts. There were a large number of frames in which no digit made contact with the pasta and these derived mainly from the guide hand, which was frequently removed and then replaced from the pasta (see above). Thus, there were three main patterns representing digit contact, one in which digits 2–5 were in contact, one in which digits 2 and 3 were in contact, and one in which only digit 2 was in contact. The relative use of the different digits for pasta holding was determined by counting digit contacts with the pasta across all grasp patterns and the results are illustrated in Fig. 4B. Digit 2 was the most frequently used digit for holding, as might be expected because it was used for both support and guide grasps, and successively less use was made of digits 3, 4 and 5, ANOVA F(3,9) = 14.8, p < 0.001 (n = 4).

**Figure 5:**
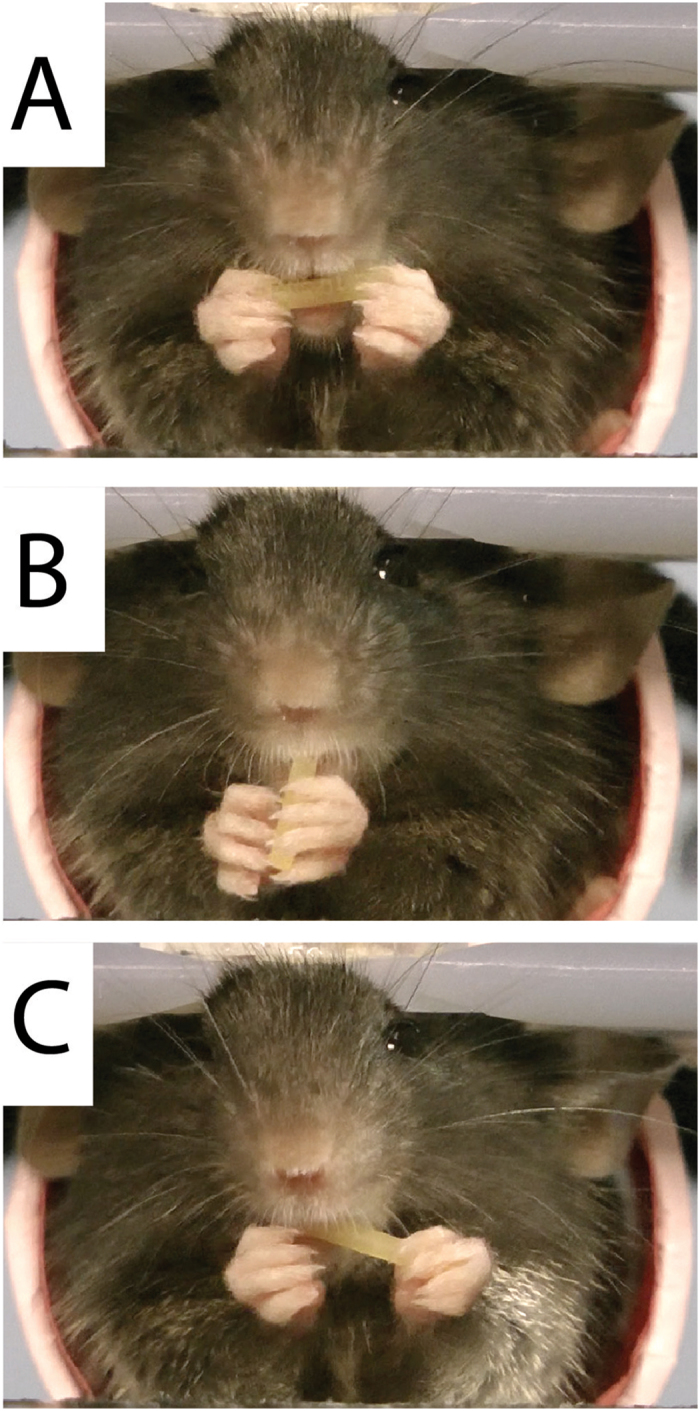
Common grasp types. (**A**) Support grasps by both the left and right hands (digits 2–3). (**B**) Support grasps by both the left (digits 2–5) and right hand (digit 2–4). (**C**) Guide grasp by the right hand (digit 2) and support grasp by the left hand (digit 2–3).

Based on the pattern and frequency of digit contacts, it was possible to identify the most commonly used grasp patterns and these are illustrated in Fig. 5. In Fig. 4A, both hands hold the pasta with digits 2–3. In Fig. 5B, the left hand holds the pasta with digits 2–5 and the right hand holds the pasta with digits 2–4. In Fig. 5C, the left hand holds the pasta with digit 2–3 and the right hand holds the pasta with digit 2.

### Relation between reach distance and hand shape

**Figure 6:**
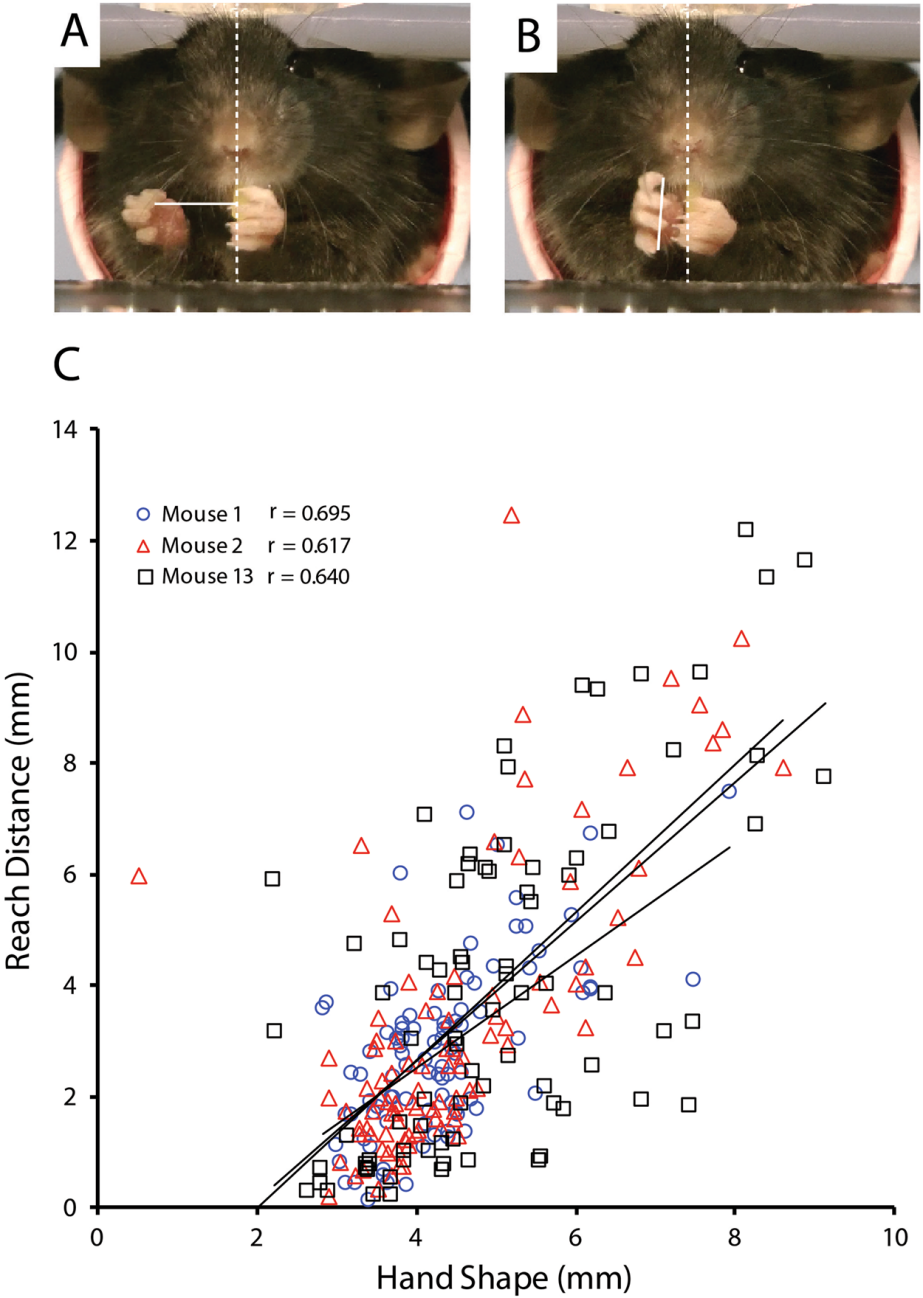
Reach distance and hand preshape size. Reach distance, the distance of the tip of digit 2 from the body midline before reach initiation (**A**, left) and hand preshape, the distance between the tip of digit 2 and the tip of digit 5 on the frame before the pasta was grasped(**B**, right) measured from 100 reaches from each of two mice. (**C**) Regression of reach distance and hand shape aperture for all reaches made by three mice each eating a single piece of pasta. Note longer reaches were associated with more open hand preshape.

To determine the relationship between reach distance and hand shape, the two were measured and correlated from 100 reaches made by each of three mice. Reach distance was measured as the furthest distance from the body midline (a plane through the center of the nose) to which the hand was abducted prior to reach initiation, as measured by the distance of digit 3 from the midline of the body (see Fig. 6A). Hand aperture was measured on the video frame before the hand made contact with the pasta as the distance between the tips of digits 2 and 5 (Fig. 6B). Figure 6C shows that there was a significant correlation between distance and aperture, Mouse 1, r = 0.70, p < 0.05; Mouse 2, r = 0.62, p < 0.05; Mouse 3, r = 0.64, p < 0.05. Thus, for all animals, a greater reach distance is associated with a more open hand shape.

## Discussion

A mouse eating a single piece of pasta makes a large number of manipulatory hand movements, nevertheless, the behaviour is organized. Eating a piece of pasta proceeds in a series of bouts, with each bout consisting of bringing the pasta to the mouth, manipulating it into a position for biting, and then chewing the piece that has been bitten from the main stem. Manipulation consists of many release-reach-grasp movements by both hands that ultimately bring the pasta to a preferred orientation for the bite. The bite is made with the molar teeth, with the pasta orientated diagonally to the contralateral lower side of the mouse. A bite is achieved by pushing the pasta downward with the support hand to break off a piece of pasta. To orient the pasta for biting, one hand usually makes a whole hand “support” grasp (with digit 2 and 3 or with combined use of digits 2,3,4, and 5) to support the pasta and the other makes a precision “guide” grasp (mainly with digit 2) to guide the pasta into the mouth. Reaches used for pasta manipulation although variable have a similar structure, with the hand advanced from a position lateral to the snout, with the digits lightly flexed and closed (collect), to a position with the digits extended and open adjacent to the pasta (overgrasp), and then to a position with the digits flexed and closed on the pasta (grasp). Longer reaches featured a larger overgrasp and made support grasps, whereas shorter reaches featured a smaller overgrasp and made guide grasps. When chewing, both hands are held in a relaxed position in front of the mouse with both often in contact with the pasta.

Beginning with Kalil and Schneider’s^5^ observation that hand postures used for spontaneous sunflower seed eating by the hamster are affected by a pyramidal tract lesion, many studies have used eating tests to assess hand posture in both rat and mouse neurological animal models of stroke, Parkinson disease, and spinal cord injury^2, 9–13^. A distinguishing feature of these studies is that they measure the static features of hand posture using rating scales based on asymmetries between an affected and an unaffected hand. The present study was directed to understanding the dynamic organization of pasta eating and the structure of the reaches associated with different grasp types. A serendipitous advantage of examining pasta-eating in head-fixed mice vs free-moving mice, is that the animal’s behaviour is more easily observed. The animal does not move around, facilitating not only the observations on an animal’s hand and mouth movements but also kinematic measures of these movements. In addition, whereas free-moving mice crouch over the food item they are eating, requiring that observations be supplemented by a mirror placed on the floor^13^, in head-fixed mice all movements are easily documented from a frontal view. The head-fixed task also shares other useful features with free-moving food eating tasks in that the mice eat pasta after a brief period of familiarization. Although the mice in the present study were trained to reach to obtain the pasta^19^, it can be just handed to an animal. In pasta-eating, a remarkably large sample of reach movements are observed and the movements are not haphazard. Within each bout of manipulating/holding the pasta, a mouse made many reach movements with each hand that varied in duration, size, and digit grasping pattern on the pasta. Nevertheless, each reach appeared to have a common structure; collect, digit preshape, grasp. This reach structure is similar to that used by mice in free-moving reaching tasks, and indeed resembles the structure of most forelimb movements that murids use for other behaviours, including stepping when walking, stepping on a horizontal ladder, and in placing responses when rearing against the wall of a cylinder^24, 25^. It is also a structure that is observed in the forelimb movements of many other species, including nonhuman and human primates^24, 25^.

The analysis of the use of the hands in support and guide roles showed that most of the mice had a preferred hand for pasta support to break off pieces of pasta, with the other hand used in a guide role. Most mice had a left hand preference but this is unlikely to mean that mice have a population asymmetry. A large sample of animals is required for forelimb preference analyses and studies with such samples have not reported that rodents display a population asymmetry that is similar to that displayed in hand use by humans^26, 27^. In addition, all of the mice had been previously trained in a reaching task that required use of their left hand, although there is no reason to believe that this training should affect this aspect of pasta eating. Furthermore, with respect to the individual asymmetry in the mice, its cause is uncertain. It may be related to a preference in use of one hand for support, a preference in the use of one hand for guiding, or it may be related to an asymmetric preference in use of one side of the mouth for biting off pieces of the pasta^28^. This feature of pasta eating could be considered in future studies.

Our novel findings of structured hand use in head-fixed mice are relevant to theoretical issues in the comparative study of hand use^29^. The variations in  hand preshapes raise the question of whether hand preshape size is related to the intrinsic (size, shape) properties of the pasta or to the functional purpose of the grasp. When humans reach to take an object from the mouth, tactile information from the mouth, and not vision, informs the hand how to preshape in relation to the intrinsic properties of the object that is to be grasped^30^. Because the pasta diameter remains constant, variations in pasta orientation and function must determine hand shape. That is, if a mouse is directing its hand to a part of the pasta close to the mouth it may use a small hand shape that ends with a guide grasp. If a mouse is directing its hand to a more distal portion of the pasta it may use a larger hand shape that ends with a guide grasp. In this respect, it is interesting that Santello *et al*.^31^ propose that hand shaping for tool use by humans although seemingly complex, involves only a few synergies. It is possible that the hand shapes displayed by the mice are synergies for guide and support functions and that this functional hand shaping is an early evolutionary step toward making hand preshapes directed mainly to the intrinsic properties of a target object.

Some features of mouse handling of pasta suggest that hand movements may be simpler than those expected of primates. First, food handling by the mice did not appear to be prospective, e.g., involving planning to move a piece of pasta from one position to another as it is eaten. Rather, it seems that it is through a series of hand and mouth movements that the pasta changes position until it obtains an orientation that allows the mouse to bite pieces from it. This manipulatory behaviour is quite different from the movements that the mice make in placing the pasta into the mouth and removing it from the mouth, both of which are clearly direct. Second, manipulation movements did not feature in-contact adjustments, in which hand and digit position is modified online to change the position of the pasta. Rather, most adjustments were made by complete hand removal followed by hand repositioning. Third, hand-shaping movements appear to be linked to reach movements such that larger reach movements were associated with larger hand preshapes. In short, mice are seemingly required to make repeated reach movements until a useful pasta position is achieved. All of this suggests that mouse reach and grasp movements are coupled and may have less prospective flexibility than do primate reach and grasp movements^24, 32^. Nevertheless, coupling of human reach and grasp movements is reported in humans reaching for objects that vary in distance^33^. The latter finding has been taken to suggest that there is some higher order control of the reach that integrates the movement in relation to distance. Perhaps the synergies of support and guide grasps favour the idea that the higher order control is an early evolutionary/rodent feature of reaching.

As reviewed by Gallistel^34^, complex behaviour, such as the pasta eating described here, likely involves two types of neural control that are hierarchically organized. A central program directs behaviour to consummation whereas a sequence of elementary units achieves that goal. As with other complex behaviour including courtship dances in birds^35^, fighting in badgers^36^, and grooming in rodents^37^, understanding the syntax of the elements simplifies what otherwise can appear to be very complex behaviour. Thus, the complexity of pasta eating is simplified by understanding that it features repeating reach units. These repeating reach units lend themselves to mesoscale neural analysis with such tools as optical imaging and optogenetics^38–41^. In each eating session, a large number of lateral to medial reaching movements can be kinematically monitored for correlations with brain activity^16, 42, 43^. The present results could also be used for neural investigations in other ways, such as at the general level of noneating vs. eating vs. chewing. It is not beyond imagination that the central program that governs pasta eating might also be reflected in mesoscale neural activity. In summary, head-fixed pasta eating is organized and features structured movements that provide the opportunity to selectively correlate neural events and hand use at a number of levels of complexity.

## Material and Methods

### Animals

Nine adult male C57/BL6 mice (2–3 months of age), weighing 20–30 g, raised at the Canadian Centre for Behavioural Neuroscience Vivarium at the University of Lethbridge, were used. These mice were previously used in Whishaw *et al*.^19^ The animals were housed in pairs under a 12:12 h light/dark cycle with light on starting at 07:30 h and temperature set at 22 °C. All testing and training was performed during the light phase of the cycle at the same time each day. The animals were placed on food restriction 3–4 days before the beginning of training and testing. The baseline weight of the mice prior to dietary restriction was recorded. The animals’ weight was monitored daily throughout the experiments to maintain body weight at 95% of prerestriction weight. To maintain body weight, mice were given additional food in their home cage within 2 h after completion of the behavioural training/testing. The animals received water *ad libitum*. The reaching success of the animals has been reported previously^19^, but not the behavioural results reported here. All experiments were carried out in accordance with the guidelines and regulations provided by Canadian Council of Animal Care and approved by the University of Lethbridge Animal Care Committee, including the guideline of reduction, minimizing the number of subjects used where possible.

### Surgical procedures

To install the head plates for the head-fixed reaching task, the animals were anesthetized with isoflurane (1 to 2%) and surgery was conducted using aseptic methods. The mice were placed in a stereotactic frame (Kopf Instruments, Tujunga, CA) on a 37–38 °C heating pad with a feedback thermistor. The animals’ eyes were covered with a thin layer of lubricating ointment (Refresh, Alergan Inc., Markham, ON). A flap of skin, approximately 1–1.5 cm^2^ was retracted from the skull, and the remaining gelatinous periostium was removed with small scissors. The skull was cleaned and dried with sterile cotton swabs. The exposed skull and skin edges were covered with a thin layer of Krazy glue, and a custom-made plastic head plate was directly affixed with the wet glue. Dental acrylic (Jet tooth, Lang Inc., Wheeling, IL) was added to cover the glue and cement the head plate in position. The head plate connected the skull rigidly to the head fixation device^44^. Animals were allowed to recover for 2–3 days before behavioural assessment.

### Video recording

Behavior was filmed with a Panasonic HDC-SDT750 camera at 60 frames per second with an exposure rate of 1ms. Illumination for filming was obtained from a two-arm cold light source, with the arms positioned to illuminate the reaching target area from a frontolateral location on each side of the mouse^19^.

### Head-fixed reaching apparatus

A custom-built head holder was used to fix the animals’ head during testing^19^. The apparatus contained two vertical side bars (3 cm long and 2.5 cm in diameter) and horizontal forks attached to a metal floor plate (20 × 18 cm) to fix the head. When the animals were head-fixed, an animals’ body, except forelimbs, were housed in a plastic tube (9 cm long and 3 cm in diameter) that oriented the body longitudinally on the same axis as the head. The tube provided support for the forelimbs while otherwise allowing relatively free movement of the shoulders and limbs. A shelf on which the food was placed was located in front of the tube. The shelf containing 9 small holes was placed before the mouse. Each hole was approximately 1.2 mm in diameter and 3 mm deep. A 1-cm long piece of pasta (spaghetti 1 mm diameter) could be placed in the holes. For the experiments, 1-cm long pieces of pasta were placed in the hole that was immediately to the reaching hand’s side of the animal’s midline. With respect to the tip of the animal’s nose, the dorsal aspect of the pasta was about 3 mm lateral, 2 mm anterior, and approximately 10 mm ventral.

### Head-fixed reaching procedure

Mice were habituated to the head-fixed apparatus for approximately 20 min per day. During this time, they were habituated to eating pieces of pasta by presenting the pasta to them with forceps. As a mouse learned to grasp with its mouth, the pasta was gradually moved further away until an animal reached for it and grasped with its hand and then grasped it from its upright position in an aperture on the shelf ^19^. Once the mice were reaching, after 3 and 4 days of training, each animal reached for up to 10 pieces of pasta each day for 15 consecutive days. Daily percent success to reach pasta was calculated by counting the number of successful reaches divided by the number of pasta pieces given in each testing session multiplied by 100. Training was considered complete once success rates reached asymptotic levels on three consecutive days of training. The improvement in success rate in the testing trials from days 1 to 15 indicated that all animals (n = 9) were able to improve their reaching skills as the testing was proceeded.

### Behavioral Procedure

**Figure 7:**
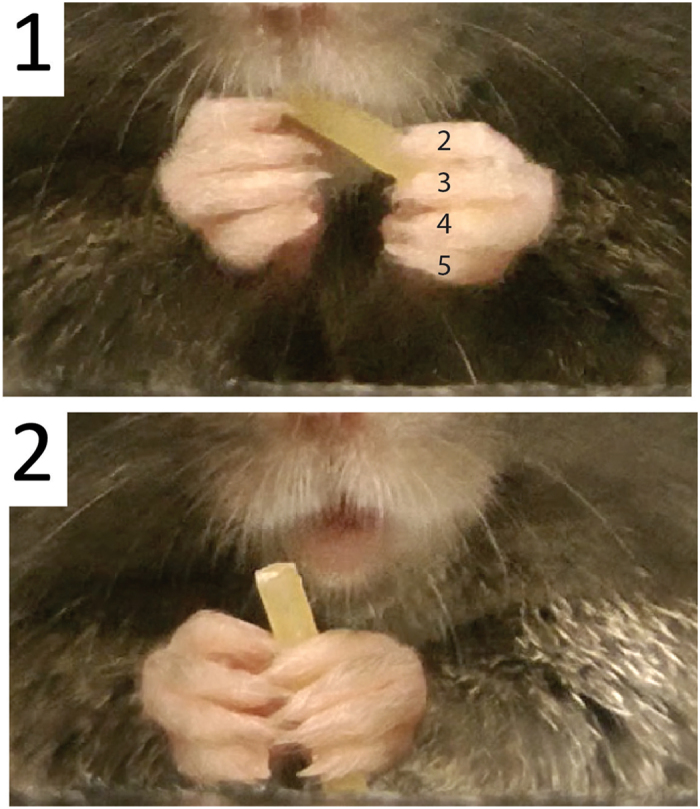
Hand posture when biting and chewing. (**A**) When biting, hands are positioned asymmetrically and hold the pasta in the mouth. The left hand digits are labeled from 2 to 5. (**B**) When chewing, hands are held together in a more symmetric posture on body midline.

The behavioral procedures were based on a conceptual framework derived from Eshkol-Wachman Movement Notation^45^, which has previously been used to describe reaching behavior in mice and other animals^1, 4, 46^. The analysis was performed by stepping frame-by-frame through the video records and with a focus on use of the hand, and digits. In the present study, the term hands rather than paws is used in keeping with previous conventions ^24^ and the digits are numbered from 1–5 with digit 1 the thumb and digit 5 the little finger (see Fig. 7). The following reaching and grasping measures were based on previous studies describing pasta eating in both rats and mice ^8, 10, 13^.

### Hand manipulation for biting and chewing

Eating pasta was characterized by periods during which an animal was actively attempting to eat the pasta and periods during which it chewed pasta that it had bitten from the main pasta piece. The acts were characterized by distinct movements and postures (Fig. 7).

- *Hand manipulation and biting*. Hand manipulation consisted of all of the hand movements that occurred as a mouse held and manipulated the pasta into a position in its mouth for biting off a piece from its end (Fig. 7A).
- *Chewing*. During chewing, the hands were lowered to a relatively symmetrical position in front of the animal with both hands closed and located proximal to, or grasping, the pasta in a relatively quiescent state (Fig. 7B).

### Reaching for and holding pasta

**Figure 8:**
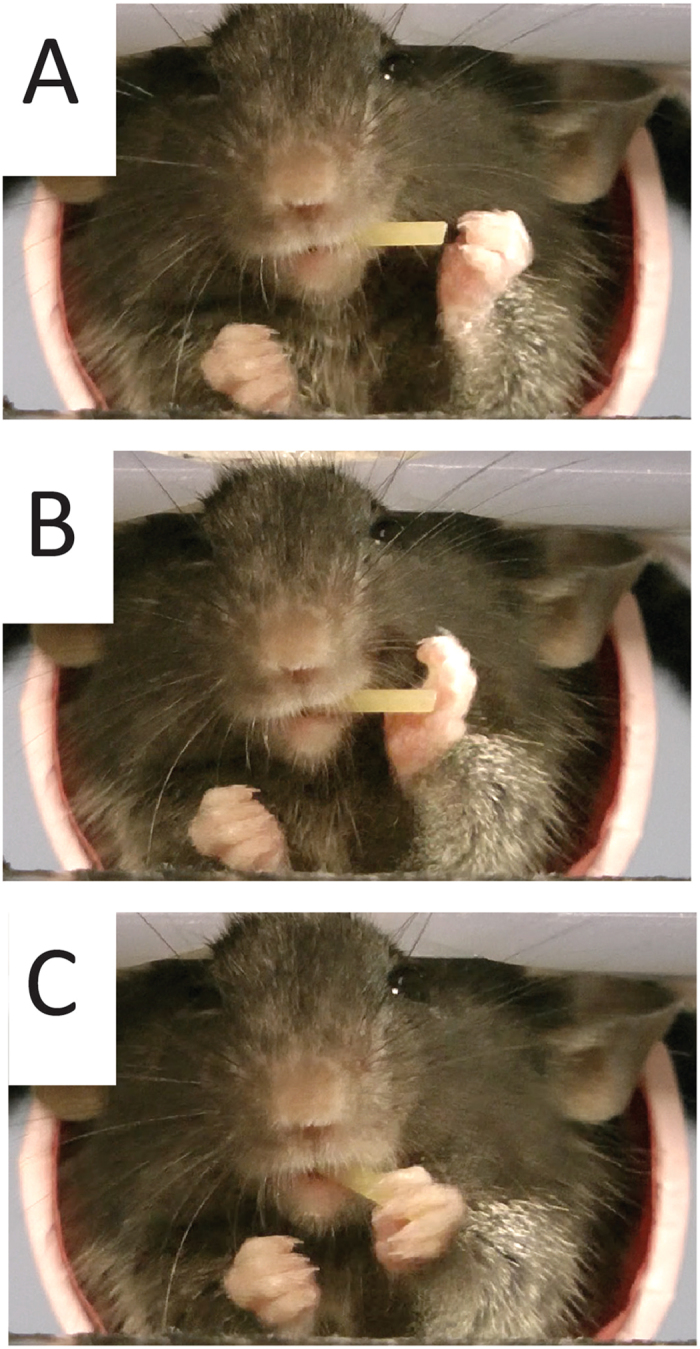
Representative hand movement during a reach. (**A**) Left hand is held in a collect posture, digits flexed and closed. (**B**) Left hand is advanced with a large hand preshape, digits extended and open. (**C**) Left hand forms a support grasp, pasta held by digit 2 and 3 and supported against the palm.

A reach was defined as a movement of a hand to grasp the pasta. A reach consisted of three hand movement components (Fig. 8):

- *Collect*. The collect posture (Fig. 8A) is one in which the digits of the hand are lightly closed and flexed and occurs before reach initiation ^25^.
- *Hand preshape*. As the hand is advanced the digits are preshaped by extending and opening in order to grasp (Fig. 8B).
- *Grasps*. As the hand contacted the pasta the digits were closed to grasp (Fig. 8C).

### Grasp types

**Figure 9:**
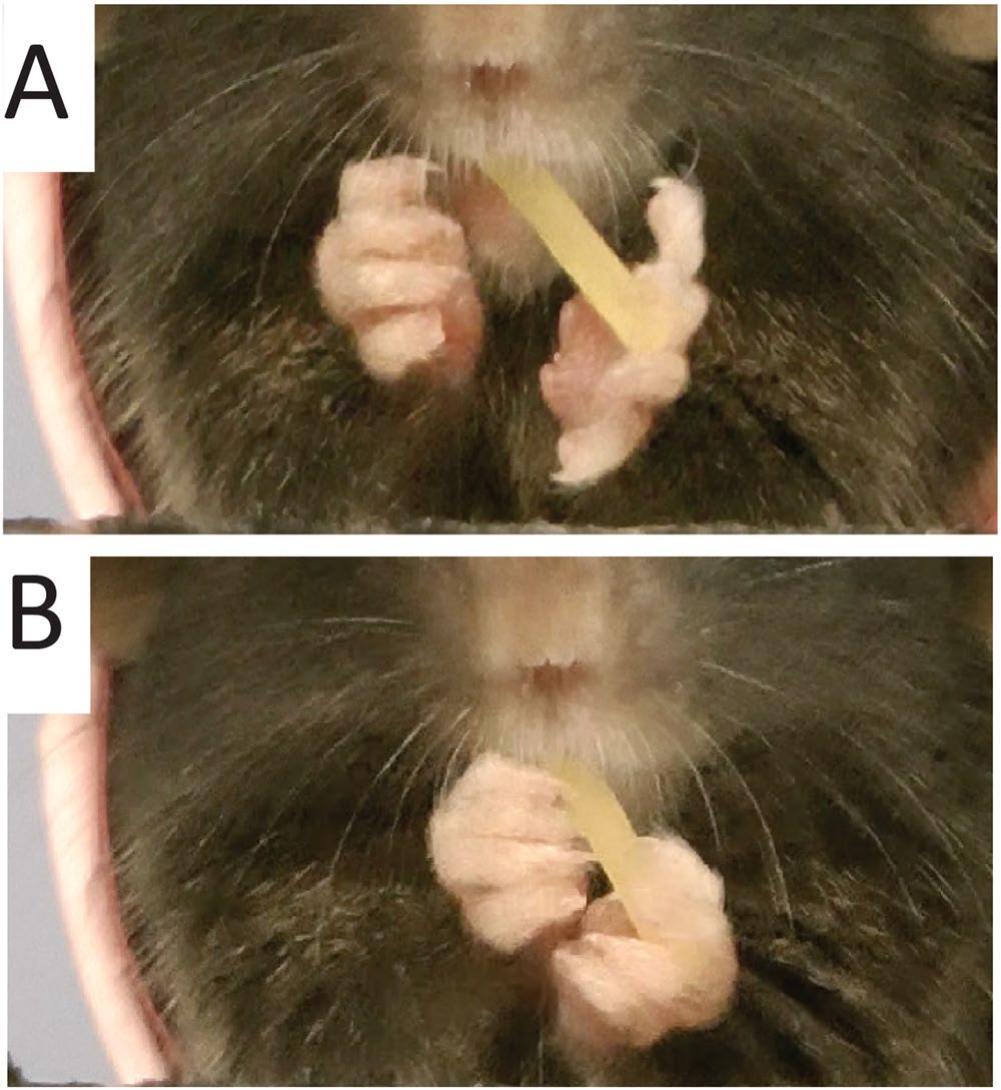
Support and guide grasps. (**A**) A guide reach features a relatively closed hand preshape and a support reach features a relatively open hand preshape. (**B**) Guide grasp made by the right hand features contact with the digit tips, is proximal to the mouth and directs the pasta into the mouth. Support grasp made by the left hand holds the pasta against the palm with digits around the pasta, is distal to the mouth, and holds for biting.

Grasps varied in terms of different patterns of digit contact with the pasta (Fig. 9) but had two functions, to support the pasta and direct it into the mouth and to guide or hold the pasta in the mouth ^22^.

- *Guide grasps*. Guide grasps directed the end of the pasta into the mouth and were generally located proximal to the mouth and featured a more closed hand preshape (Figure 9A right hand) with contact on the pasta made with digit tips (Fig. 9B, right hand).
- *Support grasps*. Support grasps were made with a more open hand preshape (Figure 9A, left hand) and involved enveloping the pasta with the digits while also pressing the pasta against the palm. They were generally located on the more distal end of the pasta (Fig. 9B, left hand).

### Pasta position and biting

The mice broke off pieces of pasta by holding one end of the pasta in the teeth and then pushing down on the other end of the pasta with a support grasp. The fragmented piece was then chewed. When the pasta broke, it made a “pop” sound that could be heard on the camera speaker. The frame on the video record on which the pasta was broken off for eating was noted from the frame-counter on the video record and then the orientation of the pasta on the frame before the break was recorded. Angle of orientation of the pasta for the bite was measured with respect to a vertical line subtending the mouse at the midpoint of its nose. The average angle of orientation was then correlated with the mouse’s handedness as defined by which hand served a support role.

### Ethogram of reaching and eating movements

Sequences of eating behavior were described by an ethogram. The ethogram was created by stepping through the video record frame-by-frame as a mouse consumed a single piece of pasta and recording behavior. Each behavior was represented by a symbol that described a component movement of reaching and eating. Symbols L, L, or l were used for left hand preshapes; symbols R, R, r for right hand preshapes, with capitalized symbols standing for an open hand preshape when reaching, capitalized and underlined symbols for a partially open hand preshape, and lowercase symbols standing for a closed hand preshape when reaching. If a reach contacted the pasta but did not succeed in grasping it, the reach was indicated with the symbol m for miss. Movements made by the guide hand that did not contact the pasta are not included in the ethogram. The movements of placing the pasta into the mouth (I), removing the pasta from the mouth (O) and chewing (C) were also noted as were reaches that contacted but missed (m) grasping the pasta. The term hand and digits is used here in keeping with the principle of a common phylogeny for skilled reaching, see Karl and Whishaw^24^.

### Digit contact when grasping

To further characterize grasp patterns, a record of digit(s) contact with the pasta was made on each of 1,000 consecutive video frames (16.6 s) from n = 4 mice. Each mouse score was obtained by a different investigator. Contacts with the digits of the left and right hand were recorded on a chart of the hand. Each digit was represented by a small circle that was checked for frames on which a digit was in contact with the pasta. Because digit 1 is small and was not visible from the front perspective, digits contacts were only recorded from digits 2, 3, 4 and 5 (from the index finger to the little finger). Although pasta was often supported against the palm of the hand, palm contribution was not analyzed because the closed digits of the hand usually obscured the pasta.

### Digitized measures of hand shape and distance

Reach distance and hand aperture for grasping were digitized from 100 reaches (n = 3 mice), each by a different investigator, using PixelStick (thepixelstick.com). A regression was performed on the rating measurements of reach distance and hand preshape.

- *Reach distance*. Reach distance was measured by digitizing the tip of digit 3 on the frame just before a reach movement was initiated (at collect) and calculating the distance to the mouse’s body midline as defined by a vertical axis running through the midpoint of the mouse’s head.
- *Hand preshape aperture*. The measure of hand preshape aperture was made by digitizing the tip of digit 2 and the tip of digit 5 on the video frame preceding hand contact with the pasta and then measuring the interdigit distance.

### Statistical analysis

The results of the study were mainly descriptive but where statistics are used, SPSS (v.19.0.0) was used for Analysis of Variance (ANOVA) and for correlations, with corrections for deviation of the distribution from normality. Error bars and ± ranges represent the s.e.m. P < 0.05, P < 0.01, P < 0.001. All the statistical tests were two-tailed.
